# Supplementary material for: Splice-Junction-Based Mapping of Alternative Isoforms in the Human Proteome
Source: Cell Rep. Author manuscript; Available in PMC 2020 Jan 15. (PMC6961840; doi:10.1016/j.celrep.2019.11.026)
Supplement: 2 [file NIHMS1546469-supplement-2.zip › DF1/PXD006675/LeftAtrium/LeftAtrium_23_NRAP_ASGDIASDLR.pdf]

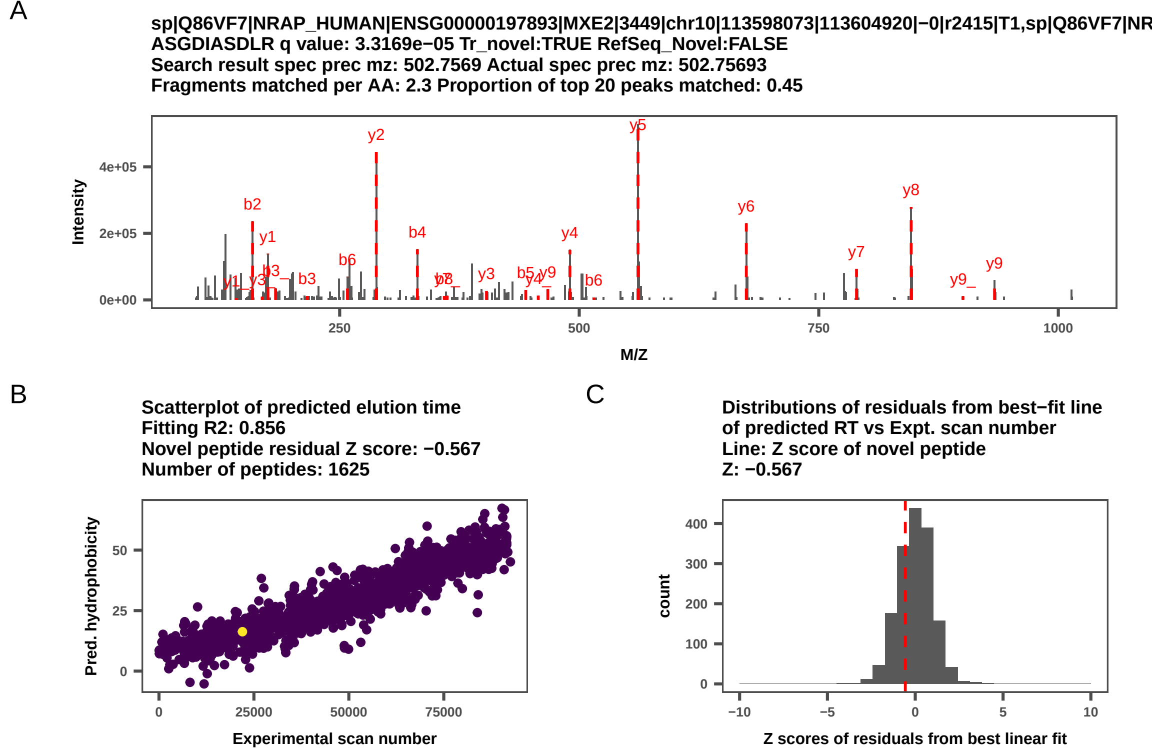
A
sp|Q86VF7|NRAP_HUMAN|ENSG00000197893|MXE2|3449|chr10|113598073|113604920|−0|r2415|T1,sp|Q86VF7|NR
ASGDIASDLR q value: 3.3169e−05 Tr_novel:TRUE RefSeq_Novel:FALSE
Search result spec prec mz: 502.7569 Actual spec prec mz: 502.75693
Fragments matched per AA: 2.3 Proportion of top 20 peaks matched: 0.45
Intensity
4e+05
2e+05
0e+00
y5
y2
b2
y1
b4
y4
y6
y8
y7
y9
b6
b3
y3
b6
y9_
250
500
750
1000
M/Z
B
Scatterplot of predicted elution time
Fitting R2: 0.856
Novel peptide residual Z score: −0.567
Number of peptides: 1625
Pred. hydrophobicity
50
25
0
0
25000
50000
75000
Experimental scan number
C
Distributions of residuals from best−fit line
of predicted RT vs Expt. scan number
Line: Z score of novel peptide
Z: −0.567
count
400
300
200
100
0
−10
−5
0
5
10
Z scores of residuals from best linear fit
